# Physicians’ Special Rate Checks

**Published:** 1884-02

**Authors:** 


### BOOK NOTICES.

PHYSICIANS' SPECIAL RATE CHECKS.—By William Jefferson Guernsey, M. D. Published by David Heston, Station F, Philadelphia.

This novelty is nothing more nor less than twenty-five checks, each with ten coupons attached, bound in receipt-book form, and to be torn out and given to patients who pay in advance for so many office prescriptions. While the plan did not at first strike us favorably, on looking further into the matter it seems really the most sensible scheme for charging "bad pay" and venereal patients especially.

TRANSACTIONS OF THE HOMŒOPATHIC MEDICAL SOCIETY OF PENNSYLVANIA, NINETEENTH ANNUAL SESSION, 1883.

The Publishing Committee are to be congratulated for the prompt and careful manner in which they issue these transactions. As for the transactions themselves, they present a goodly array of well-considered papers.

This volume illustrates the point we have commented on again and again, the tendency of "homœopathic" societies, local and general, to devote so much of their time and labors to *pathology*, excluding, as *such* work must necessarily do, to a large extent, all *original* work on our special subject, *homœopathic therapeutics.*

In the volume before us we have elaborate and lengthy papers on albuminuria, phthisis pulmonalis, and locomotor ataxia. Now, granting that the papers on these diseases have been prepared with all the learning and the labor possible, yet one must feel that little or nothing that is new or valuable has been added to the literature of these subjects. Would this have been true had the same labor been spent on our therapeutics? We believe not.

